# Fatal Anæsthetics

**Published:** 1907-06-08

**Authors:** 


### FATAL ANÆSTHETICS.

### A CASE FOR INQUIRY.

THE Coroner for Southwark, Dr. Waldo, recently held an inquest on the bodies of a child and of a woman, who died in Guy's Hospital during the administration of anæsthetics. He remarked that these complete a total of thirty such fatalities from that institution within the past six years, the last four of which occurred within a month. These figures were increased to thirty-one and five respectively by another unfortunate accident very shortly after. He is reported to have said, "In all these four cases the anæsthetics were administered, not by any of the eight anæsthetists of the hospital staff, but by other persons. Is it wise to allow men with less experience than these special anæsthetists to do this work?" It further appeared from the evidence that in the last two cases the administrators were newly qualified men holding no appointment in the hospital whatever. While in the buildings they were requested by house officers to act as stop-gap anæsthetists in order that the latter might operate.

The prominence which the above figures and Dr. Waldo's comments have received re-opens questions which have been discussed before now in the London teaching hospitals, and in which the lay press is naturally beginning to take interest. The compilation of statistics showing the fatality rates of anæsthesia in different institutions is, at first sight, quite simple; so many cases, so many deaths, such and such a ratio. But the more closely such statistics are scrutinised, the more complex their problems appear. At the present time an appreciable fraction of the cases brought to operation at the general hospitals consists of patients moribund from such serious conditions as perforated gastric and typhoid ulcers; strangulation of gut, internal or external; extensive cranial and other injuries, and so forth. While advanced surgery continues to regard no case of this sort, however desperate, as hopeless, so long must a certain proportion of deaths under anæsthesia be unavoidable, no matter how highly skilled the administration. In one general hospital, whose returns we have analysed for the past three years, about 40 per cent. of the fatalities can be fairly described under this category. Indeed, the strange thing is, not that there are so many accidents with this class of patient, but that there are so few; many of the most hopeless are operated on in the middle of the night, unprepared for anæsthesia in the technical sense, exhausted sometimes by rough and ready methods of transport to hospital and by their condition, and presenting often such obstacles to successful anæsthesia as fæcal vomiting and the like. The brands thus snatched from the burning, increasing in numbers yearly, go to the credit of the surgeon; the occasional death, though it anticipate nature by but a few hours, if it occurs "on the table" goes to the discredit of the anæsthetist.

Even some of the special hospitals have difficulties which must be taken account of in considering their records: thus the prolonged and extensive operations on debilitated and constitutionally unsound patients which form a large part of the work in the Cancer Hospital, must tell unfavourably on the operating theatre mortality there; and some others of the special hospitals have similar disabilities. Among the general hospitals also there are different proportions of out-patient and in-patient operations, of local and general anæsthesias, which all confuse the comparison of mere statistics. Another point to be remembered is that not all deaths during operations are caused by anæsthetic drugs: they are occasionally the fault of the surgeon alone, although this fact does not as a rule appear at a Coroner's inquest. We are not suggesting that this has been the case at Guy's, but undoubtedly certain deaths at other hospitals have been caused in that manner. Such cases are included in hospital reports among deaths during anæsthesia, and in the nature of things it is almost impossible to detach them from the statistics. There are yet other cases, fortunately rare, the discredit of which in no way attaches to the anæsthetist. For instance, a physician on the staff of a teaching hospital once sent an in-patient whom he was treating for lymphadenoma to the out-patient dental-room for an extraction under gas. The dental surgeon and the anæsthetist had no warning of the constitutional condition, and no blame could attach to the latter when the ensuing post-mortem revealed a trachea almost occluded by the pressure of enlarged glands; yet that case also is included among the returns of death during anæsthesia. Similarly, among the recent fatalities at Guy's Hospital, we notice that in one case an enlarged thymus and general status lymphaticus were present. This condition is so seldom diagnosed during life that it must always be a pitfall for the wariest anæsthetist, and it has, as a matter of fact, been responsible for quite a number of deaths at various London hospitals.

Before coming then, to the point especially raised by Dr. Waldo, it is evident that statistics, unless very carefully sifted, may easily lead to error. Face to face with the facts which we have quoted from the Coroner's remarks, his question is a natural one, and the answer invited is evidently a negative; but, as in all mortality statistics, there is much to be said in arrest of hasty conclusions. No doubt a system, or lack of system, which allows house officers to invite the first man they meet to administer an anæsthetic, is thoroughly unsound and dangerous to the public. No doubt a mortality of thirty-one in six years is higher than can be completely explained by the adventitious causes which we have rehearsed. We are not defending Guy's, because it seems as if something must be wrong there. Whether it is the teaching of anæsthetics, which is now rightly compulsory before a student is admited to the qualifying examinations; whether that inexperienced men are allowed to undertake difficult and dangerous cases before they have mastered the elements of the craft by practice under supervision upon easy and straightforward ones; or whether the result of any other inherent defect in the system we do not know. It may even be partly a run of unavoidable accidents that has swollen the figures of the past six years. But, in any case, there are other points to be considered before we answer the Coroner's question.

In the first place, we have examined the records of another London hospital where the average yearly number of anæsthetisations must be as great, excluding dental cases, at any rate, as at Guy's. There we must go back 12½ years to accumulate a total of 31 deaths (in about 85,000 cases). At this hospital the teaching of anæsthetic work is carried out in an exceptionally complete way, and, curiously enough, the whole of the fatalities have occurred in the practice of the regular administrators. It is, however, fair to add that probably a larger proportion of the work falls on them than in the Borough. A similar surprise appears from the records of yet a third teaching hospital, where there have been seven deaths in the past three and a half years, six of which were at the hands of the staff anæsthetists. These, no doubt, are merely a series of fortuitous coincidences, but they serve to dispel the idea that anæsthesia at the hands of well-trained hospital residents means danger to the public.

And there are yet other considerations in which the common welfare is involved. The giving of anæsthetics under supervision is very valuable training, especially after the exposition of general principles by the teacher in lectures or text-books. But such supervision must give way to independent work; the very knowledge that an expert is at hand to correct errors or deal with emergencies takes away that sense of serious personal responsibility without which no man can become a capable anæsthetist. This training, by instruction and by practice under supervision, and, lastly, by independent administrations commencing with short simple cases in out-patient and casualty rooms, and leading in the end to difficult and "emergency" work, is the kind of training which results in a large number of really capable anæsthetists leaving our London medical schools annually. An ex-house officer from a London teaching hospital who starts practice in the country is generally soon known as the best anæsthetist in his locality: indeed, the mere fact of his having held such an appointment is regarded almost of itself as a proof of his ability in anæsthetic work.

This state of things is not only gratifying to the school authorities, but also extremely important to the general public outside London. If Dr. Waldo's question is to be answered by an unqualified negative, then the better kind of general practitioner will be forced to gain his experience of anæsthesia, not in the comparatively safe way already outlined, but without help and often with imperfect appliances under the difficult conditions of private practice; a state of things which we do not think would tend to the benefit of the community. It is absurd to exalt the specialty of anæsthetic administration to the level of, let us say, gynæcology or ophthalmic surgery; some specialists, indeed, in anæsthesia aspire to stand on a footing similar to that of the consulting physician or surgeon. For the study and practice necessary to become a first-class anæsthetist bear no comparison with those required in the specialities we have mentioned. Any house officer of ordinary intelligence —be it remembered that in a teaching hospital he is one chosen out of many—after he completes his office can become a highly skilled anæsthetist, if he be not already one, in a very short time, and in a year his proficiency will be hard to distinguish from that of the elect. Moreover, it must be borne in mind that the reason of this is the excellent opportunities of which he avails himself whilst a house officer for learning the craft of anæsthetic administration. At the same time no one can realise more clearly than we do the grave danger of unskilled or partly skilled anæsthetists. We acknowledge that they exist, and that they are a reproach to the profession; but the teaching at many schools is so good, and at all it has made great strides recently, that their proportion in the profession must be steadily decreasing.

We have not touched upon the expense necessary to provide a staff of resident and visiting anæsthetists, at every hospital, large enough to cope with every possible simultaneous operation or emergency. It would certainly be no mean sum, and we doubt whether any appreciable gain in efficiency would result. Neither have we considered the effect upon the house service of a hospital which adopted regulations depriving residents of their chances of becoming familiar with this branch of practice. We think it quite possible that some of the students who do best for their hospitals as resident officers might accept appointments elsewhere, rather than forego these opportunities. So our answer to Dr. Waldo's question must be much more a qualified Yes than an unqualified No.

